# Characterization of a CTX-M-15 Producing *Klebsiella Pneumoniae* Outbreak Strain Assigned to a Novel Sequence Type (1427)

**DOI:** 10.3389/fmicb.2015.01250

**Published:** 2015-11-10

**Authors:** Kai Zhou, Mariëtte Lokate, Ruud H. Deurenberg, Jan Arends, Jerome Lo-Ten Foe, Hajo Grundmann, John W. A. Rossen, Alexander W. Friedrich

**Affiliations:** ^1^Department of Medical Microbiology, University Medical Center Groningen, University of GroningenGroningen, Netherlands; ^2^State Key Laboratory for Diagnosis and Treatment of Infectious Diseases, The First Affiliated Hospital, College of Medicine, Zhejiang UniversityHangzhou, China; ^3^Collaborative Innovation Center for Diagnosis and Treatment of Infectious DiseasesHangzhou, China

**Keywords:** *Klebsiella pneumoniae*, extended-spectrum ß-lactamase, sequence type 1427, CTX-M-15, hospital outbreak, whole-genome sequencing, polymorphism

## Abstract

Extended-spectrum -lactamase producing *Klebsiella pneumoniae* have emerged as one of the major nosocomial pathogens. Between July and September 2012, a CTX-M-15 producing *K. pneumoniae* caused an outbreak in a university hospital in the Netherlands. The outbreak isolates were characterized and assigned to a novel sequence type (ST1427). An epidemiological link between affected patients was supported by patient contact tracing and whole-genome phylogenetic analysis. Intra-strain polymorphism was detected among multiple isolates obtained from different body sites of the index patient, which may relate to antibiotic treatment and/or host adaptation. Environmental contamination caused by the outbreak clone was found in the patient rooms even on medical equipment. The novel clone was not closely related to any known endemic/epidemic clone, but carried a set of a plasmid-borne resistance genes [*bla*_CTX−M−15_, *bla*_TEM−1_, *bla*_OXA−1_, *aac(6*′*)-Ib*-cr, *qnrB1, tetA*(A), *aac(3)-II*]. Analysis of its virulence factors revealed a previously uncharacterized capsular biosynthesis region and two uncharacterized fimbriae gene clusters, and suggested that the new clone was not hypervirulent. To our knowledge, this is the first outbreak report of *K. pneumoniae* ST1427, and our study could be of help to understand the features of this newly emerging clone.

## Introduction

Extended-spectrum -lactamase (ESBL)-producing *Enterobacteriaceae* have disseminated worldwide and become a major concern for clinicians because of their limited treatment options in common infections (Paterson and Bonomo, 2005; Pitout and Laupland, 2008; Mathers et al., 2015; Tal Jasper et al., 2015). In the last decade, CTX-M-type ESBLs have replaced TEM- and SHV-type ones (Livermore et al., 2007), becoming dominant in clinical *Enterobacteriaceae* isolates. Among the CTX-M-type ESBLs, CTX-M-15 is one of the most common CTX-M-type among *Escherichia coli* isolates. Molecular epidemiological studies suggested that the global dissemination of CTX-M-15-producing *E. coli* was mainly due to a single clone (ST131) (Peirano and Pitout, 2010). Nosocomial infections caused by multidrug-resistant CTX-M-15-producing *Klebsiella pneumoniae* (CTX-M-15-KP) have dramatically increased in recent years (Lee et al., 2011; Baraniak et al., 2013; D'Andrea et al., 2013; Rodrigues et al., 2014). Different from CTX-M-15-producing *E. coli*, the population of CTX-M-15-KP is largely oligoclonal, and shows a distinctive geographical distribution. For instance, CTX-M-15-KP ST15, ST147, and ST101 are prevalent in different European countries (Baraniak et al., 2013; Rodrigues et al., 2014), whereas CTX-M-15-KP ST11 is known to have widely disseminated in Asia (Lee et al., 2011).

Conjugative plasmids are regarded as one of the main factors in the successful spread of CTX-M-type ESBLs in *K. pneumoniae* (D'Andrea et al., 2013; Mathers et al., 2015). The *bla*_CTX−M−15_ gene is often associated with specific insertion sequences (ISs) (e.g., IS*Ecp1*) and plasmids from incompatibility group F (Carattoli, 2009). It has been described that *bla*_CTX−M−3_, the presumed ancestor of *bla*_CTX−M−15_, was captured from the chromosome of *Kluyvera* spp. by IS*Ecp1*. Other mobile elements may have subsequently been involved in the movement of IS*Ecp1*-*bla*_CTX−M−15_ between plasmids and onto the chromosome of (other) members of the *Enterobacteriaceae* (D'Andrea et al., 2013).

Understanding the epidemiological and molecular features of ESBL-producing *K. pneumoniae* (ESBL-KP) population can be helpful in controlling their dissemination. In the last three decades, integration of conventional epidemiological investigation and molecular typing have greatly enhanced our knowledge on these resistant pathogens. Nowadays, whole genome sequencing (WGS) allows typing of pathogens at the highest resolution and comprehensive investigations of their molecular features (e.g., resistance mechanisms and pathogenesis). In July 2012, an outbreak of an ESBL-KP occurred in a university hospital in the north of the Netherlands. The aim of the current study was to use WGS in combination with epidemiological data to understand how the outbreak clone emerged.

## Materials and methods

### Strains collected in this study

Ten *K. pneumoniae* isolates were obtained from different clinical specimens of seven patients, of which five were related to the outbreak. Environmental sampling was performed in the patient rooms using MW728 POLYWIPE® sponge swabs (Medical wire and equipment, Wiltshire, England) and subsequent culture in brain-heart infusion mediums for 24 h. Two *K. pneumoniae* isolates obtained from the environment screening were included in this study. Strain details are listed in Table 1.

**Table 1:** ***K. pneumoniae* strains used in this study**.

**Host**	**Isolate ID**	**Sample date**	**Specimen**	**Sequence type**
Index patient	KPOI-1/1	10/07/2012	Urine	ST1427
	KPOI-1/2	20/07/2012	Urine	ST1427
	KPOI-1/3	24/07/2012	Central venous line (jugular vein)	ST1427
	KPOI-1/4	20/08/2012	Central venous line (femoral vein)	ST1427
Patient 2	KPOI-2	24/08/2012	Sputum	ST1427
Patient 3	KPOI-3	24/08/2012	Sputum	ST1427
Patient 4	KPOI-4	13/09/2012	Screening^*^	ST1427
Patient 5	KPOI-5	14/09/2012	Screening^*^	ST1427
Environment	KPEI-1	12/09/2012	Patient room (bed)	ST1427
Environment	KPEI-2	12/09/2012	Patient room (medical equipment)	ST1427
Unrelated patient	KP-11U	27/01/2012	Perineum	ST1782
Unrelated patient	KP-54M	18/10/2012	Blood	ST927

* *Specimens of rectum and throat acquired for surveillance were pooled before culturing*.

### Antimicrobial susceptibility testing

Phenotypic susceptibility testing was performed using the Vitek II system (BioMerieux, Marcy l'Etoile, France) according to the guidelines of the manufacturer and the interpretation of the breakpoints was done according to the EUCAST guidelines. In addition, an *E*-test (bioMérieux, Marcy l'Etoile, France) was performed for phenotypic confirmation of ESBL production.

### DNA extraction

DNA extraction was performed using the Ultraclean Microbial DNA Isolation Kit (MO BIO Laboratories, Carlsbad, CA, US) according to the manufacturer's instructions. The DNA concentration and purity were measured using the NanoDrop 2000c spectrophotometer (Thermo Scientific, Waltham, MA, USA) for conventional typing, and the Qubit dsDNA HS and BR assay kit (Life technologies, Carlsbad, CA, US) for WGS.

### Multilocus sequence typing (mlst)

Conventional MLST was performed using the protocol described on the *K. pneumoniae* MLST website (http://bigsdb.web.pasteur.fr). The sequence type (ST) was assigned by the MLST database (http://bigsdb.web.pasteur.fr/klebsiella/klebsiella.html). The STs of strains retrieved from GenBank and non-outbreak isolates were assigned by uploading the genomes to the webtool MLST v1.7 (https://cge.cbs.dtu.dk/services/MLST/). STs previously undescribed were submitted to the MLST database. The clonal complex analysis was performed by eBURST (http://eburst.mlst.net/).

### Whole genome sequencing, *de novo* assembly, scaffolding, and annotation

The pair-end DNA library was prepared and sequenced on the MiSeq (Illumina, San Diego, CA, USA) as described previously (Zhou et al., 2015). *De novo* assembly of the paired-end reads was performed by CLC Genomics Workbench v7.0.4 (QIAGEN, Hilden, Germany) after quality trimming (Qs ≥ 20) with optimal word sizes. Isolate KPOI-2 was randomly selected for mate-pair sequencing. The mate-pair DNA library was prepared using the Mate Pair Library Prep Kit v2 (Illumina) according to the manufacturer's instructions followed by running it on the Miseq for generating 100 bp reads. The reads were used for scaffolding the contigs generated by paired-end reads. Scaffolding was performed by SSPACE standard version 3.0 with default settings (Boetzer et al., 2011). Further gaps within scaffolds were closed using GapFiller with default settings (Boetzer and Pirovano, 2012). Genomes were manually curated by BLASTP after performing automatic annotation on the RAST server (Aziz et al., 2008) with special focus on genes of efflux pumps, fimbriae, and capsular biosynthesis.

### Single-nucleotide polymorphism (SNP) detection and core-genome phylogenetic analysis

The scaffolded genome of KPOI-2 was ordered and oriented relative to the finished genome of *K. pneumoniae* NTUH-K2044 (GenBank accession number: NC_012731) using ABACAS (Assefa et al., 2009). Reads were mapped to the rearranged KPOI-2 genome by CLC Genomics Workbench with default settings. Candidate SNPs were detected by the algorithm Quality-based variant detection of CLC Genomics Workbench. To acquire reliable SNPs, SNPs were filtered as described previously (Snitkin et al., 2012).

The detected reliable SNPs from outbreak isolates were used for SNP-based phylogenetic reconstruction by RAxML v7.4.2 (Stamatakis, 2006) with 1000 bootstrap replications under the general time-reversible model with Gamma correction (GTR+G). The core-genome phylogenetic analysis of the *K. pneumoniae* population was performed as previously described (Zhou et al., 2015). Briefly, genomes were aligned by ProgressiveMauve (Darling et al., 2010), and the core genomes were acquired by collecting fragments (≥500 bp) shared by all *K. pneumoniae* genomes analyzed here. The aligned core genomes were used for estimating the maximum likelihood (ML) phylogeny by RAxML v7.4.2 as above.

### Identification of resistance-related genes and virulence factors

The acquired antimicrobial resistance genes were identified by uploading assembled genomes to the Resfinder server v2.1 (http://cge.cbs.dtu.dk/services/ResFinder-2.1/). The other genes relating to resistance and virulence were detected using the mapping unit of CLC Genomics Workbench to map reads and/or by blasting assembled genomes to a pseudomolecule generated by concatenating a set of *K. pneumoniae* genes. The capsular genotype was determined by *wzi* typing as previously described (Brisse et al., 2013). Scaffolds with resistance-related and virulence genes were blasted against GenBank to identify their genetic location.

### Nucleotide sequence GenBank accession numbers

The Whole Genome Shotgun BioProject for our sequenced *K. pneumoniae* isolates has been deposited at DDBJ/EMBL/GenBank under the accession of JUBG00000000 (KPOI-1/1), JTKB00000000 (KPOI-1/2), JTKD00000000 (KPOI-1/3), JTKC00000000 (KPOI-1/4), JUBH00000000 (KPOI-2), JUBI00000000 (KPOI-3), JUDT00000000 (KPOI-4), JUDS00000000 (KPOI-5), JUBJ00000000 (KPEI-1), JUBK00000000 (KPEI-2), JUBL00000000 (KP-11U), JUBM00000000 (KP-54M), respectively.

## Results

### Outbreak description

**Figure 1:**
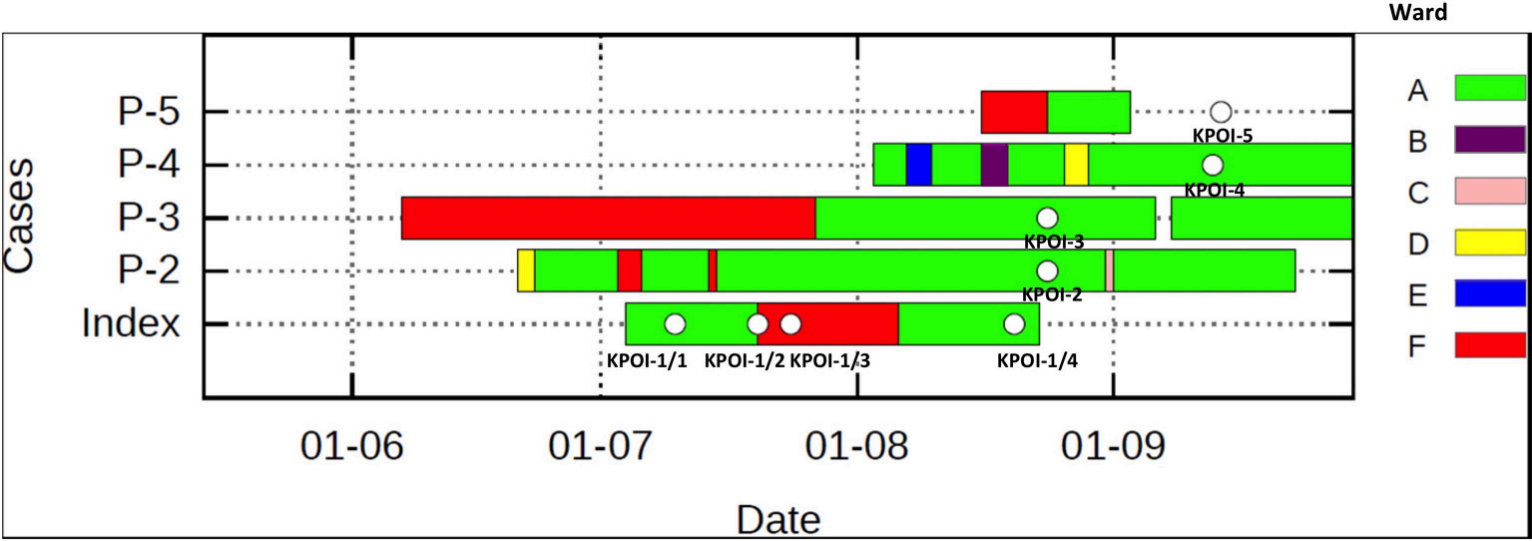
**Intra-hospital patient movements during the outbreak period**. The white spot shown on the bar represents the sequenced isolate. Patient 5 (P-5) was sampled at home after being discharged from the hospital. Different wards are indicated by different colors.

In July 2012, a patient previously hospitalized in Germany, South-Africa and Gambia was admitted to the university hospital. Due to the unavailability of a single room at the time of admission, the patient was placed in a room shared with multiple patients. Contact isolation measures were taken immediately after an ESBL-KP strain (KPOI-1/1) was cultured from the patient. After 2.5 months, regular surveillance screening (once per week) identified two ESBL-KP positive roommates of the patient. To prevent further spread, stringent infection control measures consisting of strict patient and staff cohorting were introduced. Two additional ESBL-KP positive patients were identified in the ward by contact tracing. Contact screening up to 2 weeks after the discharge of all ESBL-KP positive patients revealed no further cases and the outbreak was declared to be under control in September 2012. In total, five patients were identified of which three developed an infection (Table 1). Details of intra-hospital patient movements are shown in Figure 1.

### Antimicrobial susceptibility testing and MLST

All suspected outbreak isolates were resistant to amoxicillin, amoxicillin-clavulanic-acid, cefuroxime, cefotaxime, ceftazidime, gentamicin, tobramycin, co-trimoxazole (trimethoprim-sulfamethoxazole), ciprofloxacin, cefepime, norfloxacin, and trimethoprim and were susceptible to imipenem, polymyxin B, cefoxitin, and meropenem.

MLST analysis revealed that the five suspected outbreak isolates shared a new allelic profile (2-1-10-1-9-1-21), assigned as ST1427. eBURST analyses showed that ST1427 clustered together with ST40 and ST1704 (Figure S1). ST1427 is a single-locus variant of ST40 differing at the *phoE* locus, and is a double-locus variant of ST1704 differing at the *phoE* and *tonB* loci. This cluster has no relation with known endemic/epidemic STs as, e.g., ST11, ST14, ST15, ST23, ST86, ST101, ST147, and ST258 (Figure S1). Two non-outbreak ESBL isolates KP-11U and KP-54M belonging to a new ST (2-5-1-1-10-1-18; assigned as ST1782) and ST927 (78-9-2-1-2-1-43), respectively, were randomly selected and included in this study (Table 1).

### Core-genome phylogeny

**Figure 2:**
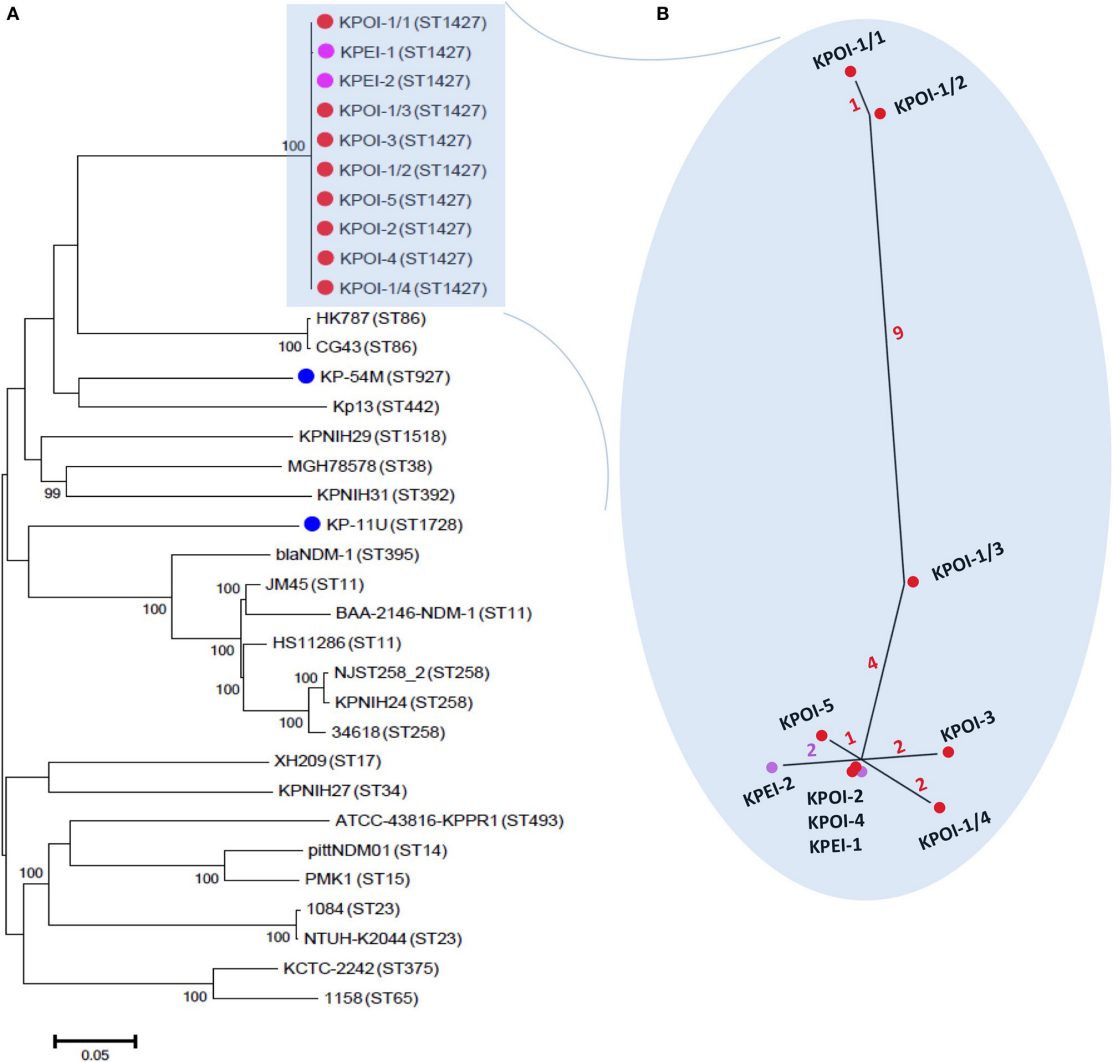
**Core-genome phylogenetic analysis of *K. pneumoniae***. **(A)** A maximum likelihood tree was constructed based on alignments of 4.4 Mb genomes which were defined as the core genomes in this study. The tree was rooted at the midpoint. The percentage of the supported bootstrap (>90) is shown. The isolates sequenced in this study are marked by dots colored red (patients involved in the outbreak), purple (environmental isolates), and blue (unrelated patients). The genomes of unmarked isolates were retrieved from GenBank. Sequence types of isolates are indicated between brackets. **(B)** The inset shows a close-up of the unrooted maximum likelihood phylogenetic tree of outbreak isolates based on 21 reliable SNPs as described in the text. The number of SNPs is indicated on the branches by different colors.

The core-genome phylogeny showed that the five suspected outbreak isolates (KPOI-1/1, KPOI-2, KPOI-3, KPOI-4, KPOI-5) clustered tightly (Figure 2A). This is concordant with the MLST results (Table 1) and available epidemiological data. To assess the genetic diversity of the new clone at the population level of *K. pneumoniae*, 22 complete genomes retrieved from GenBank (Table S1) and two ESBL-producing isolates (KP-11U and KP-45M; Table 1) obtained from our hospital representing 19 different STs were included in the phylogenetic analysis. Figure 2A shows that the outbreak clone is genetically distant from all analyzed isolates (more than 26,000 SNPs). Notably, the outbreak clone clustered with two hypervirulent ST86 K2 isolates (CG43 and HK787), suggesting that they share a common ancestor. Analyzing 120 additional *K. pneumoniae* draft genomes randomly retrieved from GenBank showed no close relation between the novel outbreak clone and any of these 120 strains (including strains belonging to ST11, ST14, ST15, ST23, ST35, ST37, ST48, ST54, ST65, ST67, ST82, ST86, ST105, ST133, ST134, ST146, ST152, ST228, ST258, ST381, ST395, ST421, ST481, ST489, ST512, ST1123, ST1125, ST1220, ST1222, ST1271, ST1272, ST1377, ST1528, ST1562, ST3751) (data not shown).

### Identification and characterization of intra-strain polymorphisms

Close inspection identified 17 SNPs among the five outbreak isolates (Figure 2B). Isolates obtained from patients 2 to 5 (KPOI-2, KPOI-3, KPOI-4, and KPOI-5) differed by 0–3 SNPs, and no SNPs were detected between KPOI-2 and KPOI-4. The isolate of the suspected index patient (KPOI-1/1) differed from the others by at least 14 SNPs. Three additional isolates (KPOI-1/2, KPOI-1/3, and KPOI-1/4) obtained from the suspected index patient were sequenced to examine whether they carried similar SNPs as KPOI-1/1. Among these three isolates, KPOI-1/4, obtained from the central venous jugular line, showed the closest relationship (2–4 SNPs) with the isolates from the other four patients. The urinary isolate KPOI-1/2 was the most distinct one differing from these four isolates by 13–15 SNPs. However, it was almost identical to the other urinary isolate KPOI-1/1 of the same patient showing only one-SNP difference. Isolate KPOI-1/3, obtained from the central venous femoral line of the index patient, showed 4–6 SNPs compared to the isolates of the other four patients, and 6–10 SNPs compared to the other three isolates of the index patient (Figure 2).

Further analysis revealed 16 unique SNPs among the index patient's four isolates (KPOI-1/1–KPOI-1/4), 10 of which were non-synonymous SNPs (NS-SNPs) (Table 2). Six of the 10 NS-SNPs were specific to the two urinary isolates (KPOI-1/1 and KPOI-1/2) (Table 2). Notably, one of the six non-synonymous SNPs was detected in the *gyrA* gene resulting in an aminoacid substitution (S83F) in the DNA gyrase. This SNP is known to confer fluoroquinolone resistance (Weigel et al., 1998). The remaining five specific NS-SNPs of KPOI-1/1 and KPOI-1/2 were mainly within genes associated with metabolism and transcriptional regulators (Table 2). Two NS-SNPs shared by KPOI-1/1, KPOI-1/2, and KPOI-1/3 were detected within a virulence-related gene *fepB* (encoding a ferric enterobactin-binding periplasmic protein), and within a gene encoding a group III Rrf2 family transcriptional regulator. The other two NS-SNPs were unique to KPOI-1/4 and were detected within genes encoding aldo-keto reductase and a hypothetical protein (Table 2).

**Table 2:** **SNPs detected among outbreak isolates of patients**.

**SNPs**	**KPOI-1/1**	**KPOI-1/2**	**KPOI-1/3**	**KPOI-1/4**	**KPOI-2**	**KPOI-3**	**KPOI-4**	**KPOI-5**	**Coding region change**	**Amino acid change**
1	C	C	T	T	T	T	T	T	FIG00642830: hypothetical protein:262A>G	Lys88Glu
2	G	A	A	A	A	A	A	A	Outer membrane protein N precursor:948T>C	Synonymous
3	C	C	C	A	A	A	A	A	^*^	^*^
4	A	A	G	G	G	G	G	G	FIG002708: Protein SirB1:245C>T	Ser82Phe
5	A	A	G	G	G	G	G	G	DNA gyrase subunit A GyrA(EC 5.99.1.3):248C>T	Ser83Phe
6	T	T	C	C	C	C	C	C	Phosphoenolpyruvate carboxylase PPC (EC 4.1.1.31):649G>A	Val217Met
7	C	C	C	T	T	T	T	T	Rrf2 family transcriptional regulator, group III:296A>G	His99Arg
8	C	C	T	T	T	T	T	T	Rrf2 family transcriptional regulator, group III:22A>G	Thr8Ala
9	T	T	C	C	C	C	C	C	^*^	^*^
10	A	A	A	G	G	G	G	G	Sugar/maltose fermentation stimulation protein homolog:657C>T	Synonymous
11	A	A	G	G	G	G	G	G	FIG00732400: hypothetical protein:96G>A	Synonymous
12	G	G	G	A	A	A	A	A	Ferric enterobactin-binding periplasmic protein FepB (TC 3.A.1.14.2):847T>C	Tyr283His
13	C	C	T	T	T	T	T	T	Transcriptional activator RfaH:377T>C	Leu126Pro
14	G	G	T	T	T	T	T	T	hypothetical protein:84A>C	
15	G	G	G	G	G	A	G	G	2-isopropylmalate synthase (EC 2.3.3.13):1517G>A	Gly506Asp
16	C	C	C	C	C	T	C	C	Transcriptional repressor of PutA and PutP:2624G>A	Gly875Asp
17	C	C	C	C	C	C	C	T	Putative HTH-type transcriptional regulator ybaO:416C>T	Ala139Val
18	A	A	A	T	A	A	A	A	Aldo-keto reductase:65T>A	Phe22Tyr
19	T	T	T	A	T	T	T	T	hypothetical protein:1049A>T	Asn350Ile

*SNPs are highlighted in red*.

* *SNPs located in intergenic regions*.

The detected SNPs were randomly distributed and no defect (i.e., mutations) was found within the DNA mismatch repair pathway (*mutS, mutH, mutL*), indicating that the intra-strain polymorphism observed in the index patient was not caused by hypermutation or recombination.

### Environment contamination

Environment sampling (*n* = 47) in the affected patient rooms resulted in the isolation of three ESBL-KP positive isolates. Isolates KPEI-1 (patient bed) and KPEI-2 (medical equipment) (Table 1) were subsequently sequenced to determine their genetic relationship with isolates from the patients. Figure 2B shows that the two environmental isolates were tightly clustered with the patient isolates. KPEI-1 was identical to KPOI-2 and KPOI-4 consistent with the fact that it was isolated from the room of patient 2. KPEI-2 showed two-SNP differences from the patients' isolates.

### The drug-resistance mechanisms

To understand the drug-resistance mechanisms of the new outbreak clone, we analyzed genes related to antimicrobial resistance.

#### Drug-resistance genes

Five genes encoding Ambler class A (TEM-1, SHV-11, two copies of CTX-M-15) and D (OXA-1) beta-lactamases were identified. The *bla*_SHV_ gene is a normal part of the chromosome in *K. pneumoniae*. In addition, resistance genes for aminoglycoside [*strAB, aac*(*6*′)-*Ib*-cr, *aac(3)-II*], fluoroquinolone [*aac*(6′)-*Ib*-cr, *qnrB1*], fosfomycin (*fosA*), sulfonamide (*sul2*), trimethoprim (*dfrA14*), tetracycline [*tetA*(A)] were found. It is known that *aac*(6′)*-Ib*-cr is a variant of the *aac*(*6*′)-*Ib* gene with reduced resistance to aminoglycosides and is able to acetylate fluoroquinolones resulting in low-level resistance. Genes *bla*_SHV−11_, *fosA* and one copy of *bla*_CTX−M−15_ were detected on scaffolds mapping to the chromosome, and the other genes were located on a scaffold mapping to a plasmid and containing an FIB replicon (Table S2). Each of the two copies of *bla*_CTX−M−15_ was found downstream of IS*Ecp1*. A 2848 bp transposition unit (IS*Ecp1*-*bla*_CTX−M−15_) flanked with 5 bp direct repeats (TAATA) was found on the chromosome. The same transposition unit was previously found in *E. coli* chromosomes in Japan (GenBank accession nr. AB683463 and AB683464) (Hirai et al., 2013). A typical 2971 bp transposition unit (IS*Ecp1*-*bla*_CTX−M−15_) was identified on the FIB plasmid. The detected resistance genes are fully concordant with the phenotypically derived resistance profile.

#### Outer membrane porins

Genes *ompK35* and *ompK36* encoding two major outer-membrane porins were identified in the outbreak strain (Table S2). Neither *ompK35* nor *ompK36* had any obvious changes that would be expected to alter their expression or function.

#### Multidrug resistance (MDR) efflux pumps

Numerous MDR efflux pumps were found to be present in the outbreak strain (Table S2). AcrAB-TolC is one of the ubiquitous efflux pumps of the resistance nodulation division (RND) family in *Enterobacteriaceae*, and mutations in its regulators *acrR, marR, ramR*, and *soxR* enable *acrAB* to be overexpressed resulting in a MDR phenotype (Bialek-Davenet et al., 2011). We did not find any mutations in these regulators. In addition, the recently described RND efflux pump KexD was identified. This efflux pump is associated with resistance against erythromycin, tetracycline, novobiocin, and some dyes (Ogawa et al., 2012), and is not ubiquitously present in *K. pneumoniae* (Li et al., 2014).

### The pathogenicity

The pathogenicity of the new outbreak clone was analyzed by searching the various virulence factors.

#### Adhesins

Fimbriae are one of the major adhesins of *K. pneumoniae*. Ten different fimbriae gene clusters were identified (Table S3). An *E. coli* common pilus (ECP) (also known as Mat pilus) was detected on the chromosome of the outbreak strain. Like type 1 and 3 fimbriae, ECP also contributes to the colonization and biofilm formation resulting in enhanced virulence of *K. pneumoniae* (Alcántar-Curiel et al., 2013). Besides five of seven fimbriae (Kpa, Kpd, Kpe, Kpg, and Kpf) previously identified in the hypervirulence *K. pneumoniae* strain NTUH-2044, two uncharacterized fimbriae gene clusters were identified, named Kph and Kpi in this study. Additionally, a polysaccharide adhesin encoded by the *pgaABCD* operon was detected, which is involved in the enhancement of biofilm formation, intestinal colonization, extraintestinal dissemination, and induction of systemic infection (Chen et al., 2014).

#### Capsule

**Figure 3:**
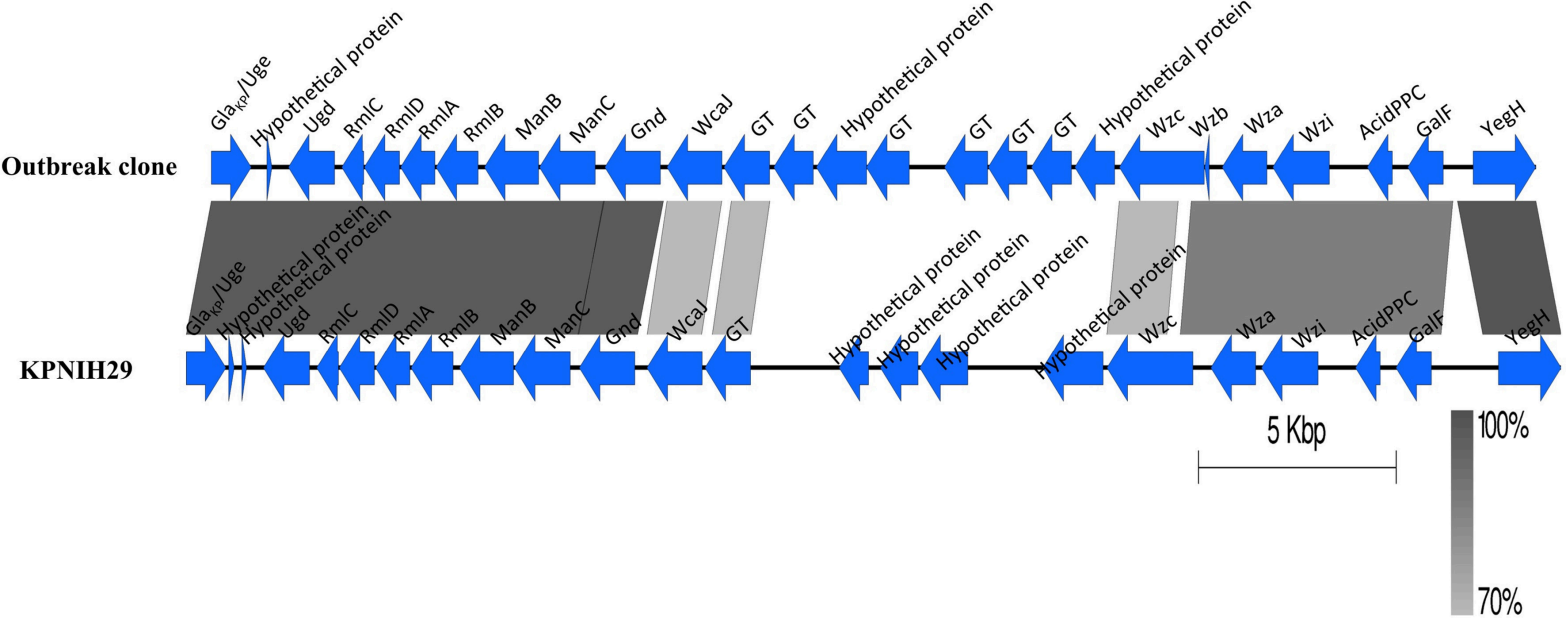
**Comparison of the capsular polysaccharide synthesis (*cps*) region**. The *cps* region (including conserved flanking region) of *K. pneumoniae* KPNIH29 (GenBank accession number: CP009863) is retrieved from GenBank. The gradients (dark to pale) of the alignment region represent the percentage of sequence identity between samples as defined by BLASTn. GT represents glycosyltransferase.

An uncharacterized capsular polysaccharide biosynthesis region (*cps*) was identified (Figure 3). The best hit in GenBank is *K. pneumoniae* strain KPNIH29 (GenBank accession number: CP009863) with a 57% coverage and 97% identity. The *wzi* typing showed that the capsule is closely related to genotype *wzi*-73 but contained two SNPs (T411C and T414C). Further analysis of the *cps* variable region (between the *wzc* and *wcaJ* genes) by blasting against the database “Whole-genome draft contigs” revealed a unique match with *K. variicola* strain KVR801v1 (GenBank accession number: CDMV01000000).

#### Iron uptake systems

Iron uptake is essential for bacterial growth and important for its virulence. Seven common iron uptake systems belonging to four major classes were identified: ABC transporter (Sit, Fec, and Eit), ferrous transporter (Feo), hemophore-based uptake system (Hmu), and siderophore-based uptake systems (Fep-Ent and Fhu) (Table S3). Only a single chromosomal gene was detected for IroA (*iroN*) and Iuc (*iutA*) siderophore-based uptake systems, both of which are highly associated with hypervirulent *K. pneumoniae* (Li et al., 2014).

#### Nitrogen utilization systems

The outbreak clone harbored a cytoplasmic urease biosynthesis operon *ureDABCEFG*, which is able to enhance the growth of *K. pneumoniae* in the host (e.g., in the gastrointestinal and urinary tract) by hydrolyzing urea to ammonia and carbon dioxide.

#### Secretion systems

Besides type I and II secretion systems (T1SS, T2SS) (data not shown), two intact but different T6SS were identified on the chromosome of the outbreak clone (Figure S2). T6SS enables the secretion of toxins using a needle-like mechanism, and also plays a role in interbacterial antagonism and biofilm formation (Bonemann et al., 2010; Russell et al., 2014). An F-like T4SS was found on an ESBL-encoding IncFIB plasmid (Figure S3), which is mainly involved in DNA transfer by plasmid conjugation (Lawley et al., 2003). TraT of the T4SS was interrupted by a mobile element protein (Figure S3). The detected T4SS was highly similar to the one of plasmid p6234–198.371 kb (GenBank accession nr. CP010390) with 98 identity and 95% coverage, which also carried a *bla*_CTX−M−15_ gene. Differences were mainly found in surrounding regions of TraT.

## Discussion

In this communication, we used WGS to study a CTX-M-15 producing *K. pneumoniae* outbreak clone, assigned to a new sequence type (ST1427). Not surprisingly, WGS allowed a higher typing resolution compared to conventional typing methods, such as MLST. Whole-genome SNP analysis revealed that the first isolate KPOI-1/1 was significantly different from the other outbreak isolates. This may have been caused by either: (i) the index patient carried various *K. pneumoniae* mutants and KPOI-1/1 was not the real index isolate; (ii) KPOI-1/1 was hypermutable; or (iii) the index patient was not correctly identified in this outbreak. To clarify the reason, three additional isolates obtained from different body sites of the index patient were analyzed. Isolate KPOI-1/4 obtained from the central venous jugular line was suggested to be the most likely index isolate of the outbreak as it showed the closest relationship (2–4 SNPs) with the isolates from the other patients. The intra-strain polymorphism observed in the index patient indicates that inclusion of multiple isolates from a single patient, especially of a suspected index patient, can be helpful for drawing proper conclusions during outbreak management using SNP-based typing methods. This also indicates that highly similar clones presenting with different drug-resistance patterns may exist in a single patient, which may have impact on patient management, e.g., prescription of antibiotics.

Our further analyses excluded recombination and hypermutation as the driven force for the observed intra-strain polymorphism among the index patient's isolates. The intra-strain polymorphism might not completely be caused spontaneously but may be associated with selective pressures, e.g., antibiotic treatment and/or host adaptation. This is supported by the observation that most detected NS-SNPs were located within genes associated with metabolism/virulence, transcriptional regulation and antibiotic resistance (Table 2). Especially, a known NS-SNP causing fluoroquinolone resistance due to an aminoacid mutation in the DNA gyrase (S83F) was exclusively detected in the two almost identical urinary isolates (KPOI-1/1 and KPOI-1/2) of the index patient, who received ciprofloxacine treatment during admission. Therefore, fluoroquinolones may have been involved in causing the intra-strain polymorphism found in the isolates of the index patient. The other five unique NS-SNPs (Table 2) shared by the two urinary isolates may be of advantage to the bacterium during urinary tract infection/colonization, as the urinary tract is full of various stress factors including mechanical shear stress, host immune responses, limitation of iron, nutrients and oxygen, and antibiotic treatment (Tielen et al., 2013). A similar observation of the intra-strain diversity has recently been reported in 22 morphologically identical *Pseudomonas aeruginosa* isolates obtained from a single Cystic Fibrosis patient (Darch et al., 2015). In addition, it has also been suggested that long-term colonization may cause clone diversity (Yang et al., 2011). Although not enough samples were available to resolve this, it's notable that the index patient had been hospitalized in Germany, South Africa, and Gambia for long periods, before being admitted to our hospital.

The new outbreak clone was not related to any known endemic/epidemic clones. However, a set of a plasmid-borne resistance genes [*bla*_CTX−M−15_, *bla*_TEM−1_, *bla*_OXA−1_, *aac(6*′*)-Ib*-cr, *qnrB1, tetA*(A), *aac(3)-II*] was identified on the plasmid, known to circulate in *K. pneumoniae* (Dolejska et al., 2013; Filippa et al., 2013; Huang et al., 2013; Bialek-Davenet et al., 2014) and other *Enterbacteriaceae* strains (Machado et al., 2006) disseminated throughout Europe. Acquisition of this resistome makes bacteria resistant to antibiotics frequently used within the healthcare system, thereby increasing the risk of causing outbreaks. Moreover, these genes may easily be transferred to other bacteria via plasmid transfer as an intact *tra* region was found, contributing to further dissemination of these genes. Indeed, blasting the scaffold of the plasmid suggested that part of the plasmid shared high similarity with some plasmids carrying the same resistome (e.g., GenBank accession nr. CP010390). Therefore, active surveillance of such epidemic resistome/plasmid may be helpful in preventing further dissemination of the resistant clones.

Analysing the virulence factors of the outbreak clone revealed the lack of multiple virulent factors frequently associated with hypervirulent *K. pneumoniae* strains, e.g., the K1/K2 capsular serotype, yersiniabactin, aerobactin, salmochelin, allantoin metabolism, and a hypermucoviscous phenotype (caused by a *rmpA* gene) (Shon et al., 2013). Remarkably, the outbreak clone carried an uncharacterized *cps* region, and its *wzi* genotype was highly similar to genotype *wzi*-73 as determined by *wzi* typing (Brisse et al., 2013). In *K. pneumoniae*, the *cps* region is very diverse, comprising eight conserved CDSs at the 5′ (*galF, orf2, wzi, wza, wzb, wzc*) and 3′ end (*gnd* and *ugd*), and a variable region in between (Shu et al., 2009). Analysis of the GC content suggests that the evolutionary origins of the variable regions are distinct from the conserved CDSs caused by homologous recombination (Wyres et al., 2015). The uncharacterized *cps* region shared a similar variable region with a *K. variicola* strain, indicating a recombination event may have occurred between the outbreak clone and the *K. variicola* strain. This feature enables us to design an outbreak-specific PCR for rapid patient screening during future outbreaks with this clone.

In summary, our study shows that analysing the genetic features of this novel outbreak clone in relation to its resistance and pathogenicity may be helpful for patient management and outbreak surveillance in hospital settings.

## Author contributions

KZ, ML, RD, JA, JL, HG, JR, and AF participated in the design and/or discussion of the study. KZ and ML carried out the major experiments. KZ, ML, and RD analyzed the data. KZ, ML, and JR wrote the paper. JL, HG, JR and AF revised it for important intellectual improvement. All authors read and approved the final version to be published.

## Funding

This study was partly supported by the Interreg IVa-funded projects EurSafety Heath-net (III-1-02=73) and SafeGuard (III-2-03=025) and by a University Medical Center Groningen Healthy Aging Pilots grant.

### Conflict of interest statement

The authors declare that the research was conducted in the absence of any commercial or financial relationships that could be construed as a potential conflict of interest.

## Supplementary material

Table S1 ***K. pneumoniae* strains retrieved from GenBank with complete genome sequence included in this study**.

Table S2 **Genes related with drug resistance identified in the outbreak clone**. ^#^Only 1 synonymous SNP was detected. ^‡^The best hit is defined by Blastn on http://blast.ncbi.nlm.nih.gov/Blast.cgi. All coverages are 100%.

Table S3 **Virulence factors of the outbreak clone**. ^*^The best hit is defined by Blastn on http://blast.ncbi.nlm.nih.gov/Blast.cgi ^†^ These unidentified fimbriae are named in this study, and the loci of the best hit are used here as gene names.

Figure S1 **Population snapshot of *K. pneumoniae***. The population snapshot was determined by eBURST analysis (http://eburst.mlst.net/), showing the relationship of sequence types in the *K. pneumoniae* MLST database (http://bigsdb.web.pasteur.fr/klebsiella/klebsiella.html). The new sequence type ST1427 detected in this study is indicated.

Figure S2 **Comparison of the T6SS loci identified in the outbreak clone and NTUH-K2044**. The gradients (dark to pale) of the alignment region represent the percentage of sequence identity between samples defined by BLASTn. The *clpV* gene of NTUH-K2044-Locus II and KPOI-Locus II is not shown in the figure since it is present on a different locus.

Figure S3 **The *tra* region of the T6SS loci identified in the outbreak clone**. The upper cases represent Tra proteins (e.g., A, TraA; B, TraB), and the lower cases represent Trb proteins (e.g., c, TrbC; f, TrbF).
