# Supplementary material for: Characterization of a CTX-M-15 Producing Klebsiella Pneumoniae Outbreak Strain Assigned to a Novel Sequence Type (1427)
Source: Front Microbiol. 2015 Nov 10;6:1250. doi: 10.3389/fmicb.2015.01250 (PMC4639626; doi:10.3389/fmicb.2015.01250)
Supplement: Table S1 — K. pneumoniae strains retrieved from GenBank with complete genome sequence included in this study. [file DataSheet1.DOCX]

**Supplementary material**

**Table S1 *K. pneumoniae* strains retrieved from GenBank with complete genome sequence included in this study**

| **Isolate** | **ST** | **GenBank accession number** |
| --- | --- | --- |
| KPNIH24 | ST258 | CP008797 |
| 34618 | ST258 | CP010392 |
| 30684/NJST258_2 | ST258 | CP006918 |
| KPNIH31 | ST392 | CP009876 |
| KPNIH29 | ST1518 | CP009863 |
| KPNIH27 | ST34 | CP007731 |
| CG43 | ST86 | NC_022566 |
| HK787 | ST86 | CP006738 |
| blaNDM-1 | ST395 | CP009114 |
| ATCC 43816 KPPR1 | ST493 | CP009208 |
| MGH 78578 | ST38 | NC_009648 |
| PMK1 | ST15 | CP008929 |
| pittNDM01 | ST14 | CP006798 |
| 1158 | ST65 | CP006722 |
| 1084 | ST23 | NC_018522 |
| NTUH-K2044 | ST23 | NC_012731 |
| XH209 | ST17 | CP009461 |
| Kp13 | ST442 | CP003999 |
| KCTC-2242 | ST375 | NC_017540 |
| JM45 | ST11 | NC_022082 |
| HS11286 | ST11 | NC_016845 |
| ATCC BAA-2146 | ST11 | CP006659 |

**Table S2 Genes related with drug resistance identified in the outbreak clone**

| **Gene Category** | **Genetic location** | **Resistance phenotype** | **Best Hit^‡^ (GenBank accession No.)** |
| --- | --- | --- | --- |
| **Drug-resistance gene** |  |  |  |
| *bla*_TEM-1_ | Plasmid | Penicillins, narrow-spectrum cephalosporins, inhibitor-sensitive | 100% (JF910132) |
| *bla*_OXA-1_ | Plasmid | Penicillins, inhibitor-resistant | 100% (J02967) |
| *bla*_SHV-11_ | Chromosome | Penicillins, narrow-spectrum cephalosporins, inhibitor-sensitive | 99%^#^ (KF585136) |
| *bla*_CTX-M-15_ | Plasmid and chromosome | Penicillins, extended-spectrum cephalosporins, aztreonam | 100% (DQ302097) |
| *strAB* | Plasmid | Streptomycin | 100% (AF321551, M96392) |
| *aac(6')-Ib-*cr | Plasmid | Aminoglycosides, fluoroquinolones | 100% (DQ303918) |
| *aac(3)-II* | Plasmid | Gentamicin, tobramycin, netilmicin, sisomicin | 100% (JX424423) |
| *qnrB1* | Plasmid | Quinolones, fluoroquinolones | 100% (NG_036203.1) |
| *sul2* | Plasmid | Sulfonamides | 100% (GQ421466) |
| *dfrA14* | Plasmid | Trimethoprim | 100% (GU726917) |
| *fosA* | Chromosome | Fosfomysin | 99% (CP009114) |
| *tetA*(A) | Plasmid | Tetracyclines | 100% (AJ517790) |
| **Porin** |  |  |  |
| *ompK35* | Chromosome | Cephalosporins and carbapenems | 99% (CP003999) |
| *ompK36* | Chromosome | Cephalosporins and carbapenems | 99% (FO203501) |
| **Efflux pump (familiy)** |  |  |  |
| *acrAB-tolC* (RND) | Chromosome | Aminoglycosides, beta-lactams, tigecycline, macrolides | 99% (NC_012731) |
| *acrD* (RND) | Chromosome | Aminoglycosides, deoxycholate, fusidic acid, novobiocin | 99% (CP003999) |
| *kexD* (RND) | Chromosome | erythromycin, tetracycline, novobiocin, dyes | 99% (CP003999) |
| *mdtABC* (RND) | Chromosome | Deoxycholate, novobiocin, bile salt | 99% (CP006659) |
| *oqxAB* (RND) | Chromosome | Chloramphenicol, fluoroquinolones, trimethoprim | 99%/100% (CP009461) |
| *eefABC* (RND) | Chromosome | Chloramphenicol, tetracyclines, ciprofloxacin | 99% (CP006659) |
| *bcr* (MF) | Chromosome | Bicyclomycin, sulfathiazole | 99% (CP003999) |
| *emrAB* (MF) | Chromosome | Nalidixic acid, hydrophobic compounds | 99% (NC_012731) |
| *fsr* (MF) | Chromosome | Fosmidomycin | 99% (CP003999) |
| *mdfA (kdeA)* (MF) | Chromosome | Aminoglycosides, fluoroquinolones, chloramphenicol | 99% (CP003999) |
| *mdtG* (MF) | Chromosome | Deoxycholate, fosfomysin | 99% (CP006659) |
| *mdtH* (MF ) | Chromosome | Enoxacin, norfloxacin | 99% (CP006659) |
| *mdtL* (MF) | Chromosome | chloramphenicol | 99% (CP006659) |
| *smvA*(MF) | Chromosome | Acriflavine, quaternary ammonium compounds | 100% (CP003999) |
| *sugE* (SMR) | Plasmid and Chromosome | benzalkonium chloride, ethidium bromide | 100% (CP000650, CP010361) |
| *mdtK* (MATE) | Chromosome | acriflavine, norfloxacin | 99% (CP006722) |
| *macAB-tolC* (ABC) | Chromosome | Macrolides | 99% (NC_012731) |

^#^Only 1 synonymous SNP was detected.

‡The best hit is defined by Blastn on http://blast.ncbi.nlm.nih.gov/Blast.cgi. All coverages are 100%.

**Table S3 Virulence factors of the outbreak clone**

| **Category of virulence factor** | **Gene(s) detected** | **Best hit^*^(coverage, identity)** | **Genetic location** |
| --- | --- | --- | --- |
| **Adhesin** |  |  |  |
| Type 1 fimbriae | *fimBEAICDFGHK* | CP009863 (100%, 99%) | Chromosome |
| Type 3 fimbriae | *mrkABCDF* | CP010361 (100%, 99%) | Chromosome |
| Kpa fimbriae | *kpaABCDE* | CP009461 (100%, 99%) | Chromosome |
| Kpd fimbriae | *kpdRABCD* | CP009863 (100%, 99%) | Chromosome |
| Kpe fimbriae | *kpeABCD* | CP006798 (100%, 99%) | Chromosome |
| Kpf fimbriae | *kpfRABCD* | NC_009648 (100%, 99%) | Chromosome |
| Kpg fimbriae | *kpgABCD* | CP009461 (100%, 99%) | Chromosome |
| Kph fimbriae^ǂ^ | locus: KPNIH31_02745-02765 | CP009876 (100%, 99%) | Chromosome |
| Kpi fimbriae*^ǂ^* | locus: FH42_19045-19060 | CP009114 (100%, 99%) | Chromosome |
| ECP (Mat) fimbriae | *ecpRABCDE* | FO834906 (100%, 99%) | Chromosome |
| polysaccharide adhesin | *pgaABCD* | NC_017540 (100%, 99%) | Chromosome |
| **Capsule** |  |  |  |
| New genotype | Fig. 3 | CP009863 (57%, 97%) | Chromosome |
| **Iron uptake system** |  |  |  |
| Fep-ent (Enterobactin) | *fepA-entD, fes-entF, fepDGC, ybdA, fepB, entCEBA* | CP009876 (100%, 99%) | Chromosome |
| Iuc (Aerobactin) | *iutA* | CP006648 (100%, 99%) | Chromosome |
| IroA (Salmochelin) | *iroN* | CP003785 (100%, 100%) | Chromosome |
| Fhu (Ferrichrome) | *fhuACDB* | CP006648 (100%, 99%) | Chromosome |
| ABC transporter Sit | *sitABCD* | CP009461 (100%, 99%) | Chromosome |
| ABC transporter (Iron/B12/siderophore/hemin) | *eitABCD* | CP009863 (100%, 99%) | Chromosome |
| ABC transporter Fec (Ferric dicitrate) | *fecIRABCDE* | NC_023333 (100%, 99%) | Plasmid |
| Feo (Ferrous iron transport) | *feoABC* | CP006648 (100%, 99%) | Chromosome |
| Hmu (Hemin/hemoprotein) | *hmuRSTUV* | NC_012731 (100%, 99%) | Chromosome |
| **Nitrogen source utilization** |  |  |  |
| Urease | *ureDCBAEFG* | NC_017540 (100%, 99%) | Chromosome |
| **Secretion system** |  |  |  |
| T4SS | Fig. S3 | CP010390 (95%, 98%) | Plasmid |
| T6SS | 2 copies (Fig. S2) | NC_017540 (100%, 99%); CP009863 (99%, 99%) | Chromosome |

**^*^**The best hit is defined by Blastn on http://blast.ncbi.nlm.nih.gov/Blast.cgi.

^ǂ^These unidentified fimbriae are named in this study, and the loci of the best hit are used here as gene names.
