# Supplementary figures and images for: Characterization of a CTX-M-15 Producing Klebsiella Pneumoniae Outbreak Strain Assigned to a Novel Sequence Type (1427)
Source: Front Microbiol. 2015 Nov 10;6:1250. doi: 10.3389/fmicb.2015.01250 (PMC4639626; doi:10.3389/fmicb.2015.01250)

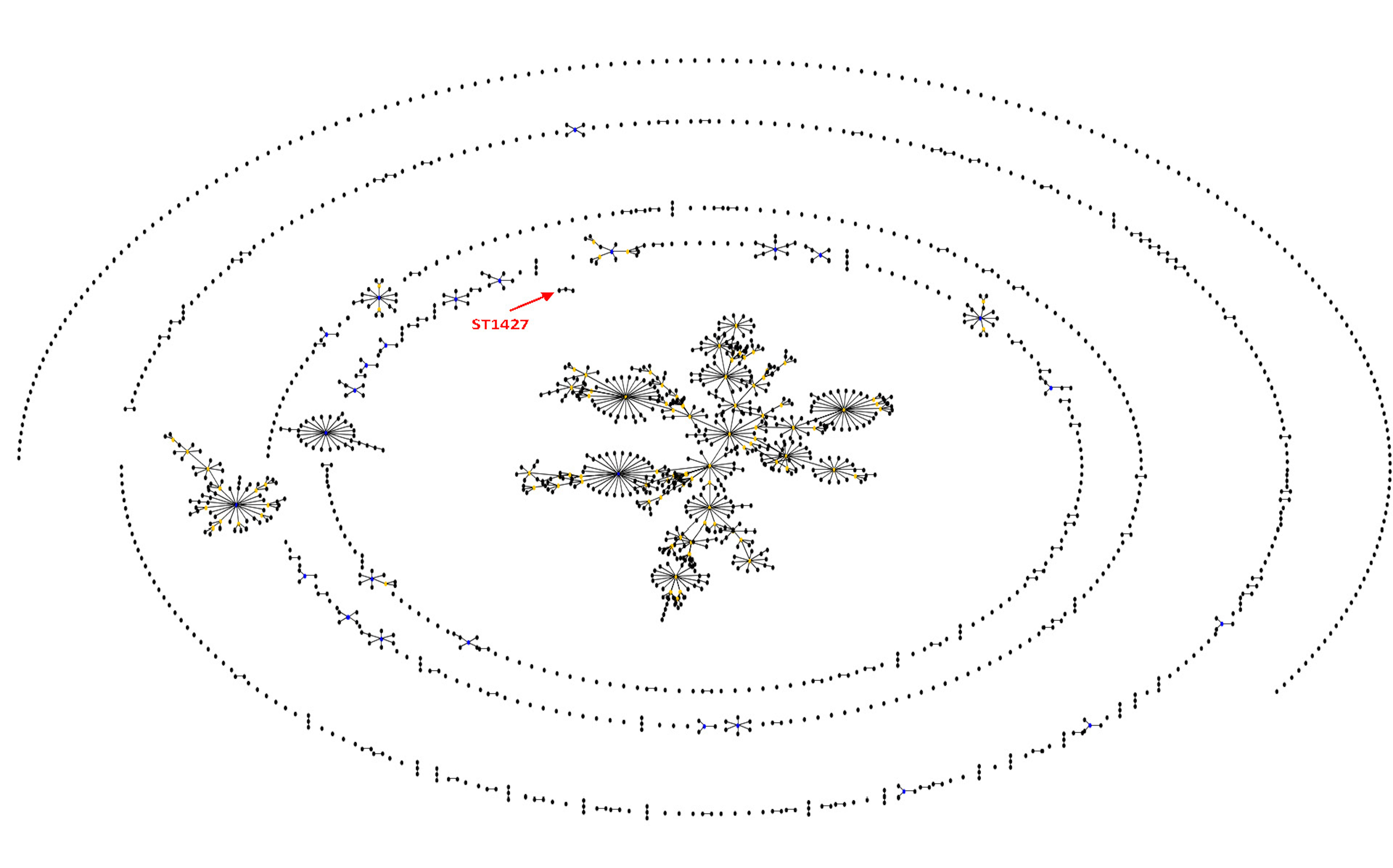

Supplement: Figure S1 — Population snapshot of K. pneumoniae. The population snapshot was determined by eBURST analysis (http://eburst.mlst.net/), showing the relationship of sequence types in the K. pneumoniae MLST database (http://bigsdb.web.pasteur.fr/klebsiella/klebsiella.html). The new sequence type ST1427 detected in this study is indicated. [file Image1.JPEG]

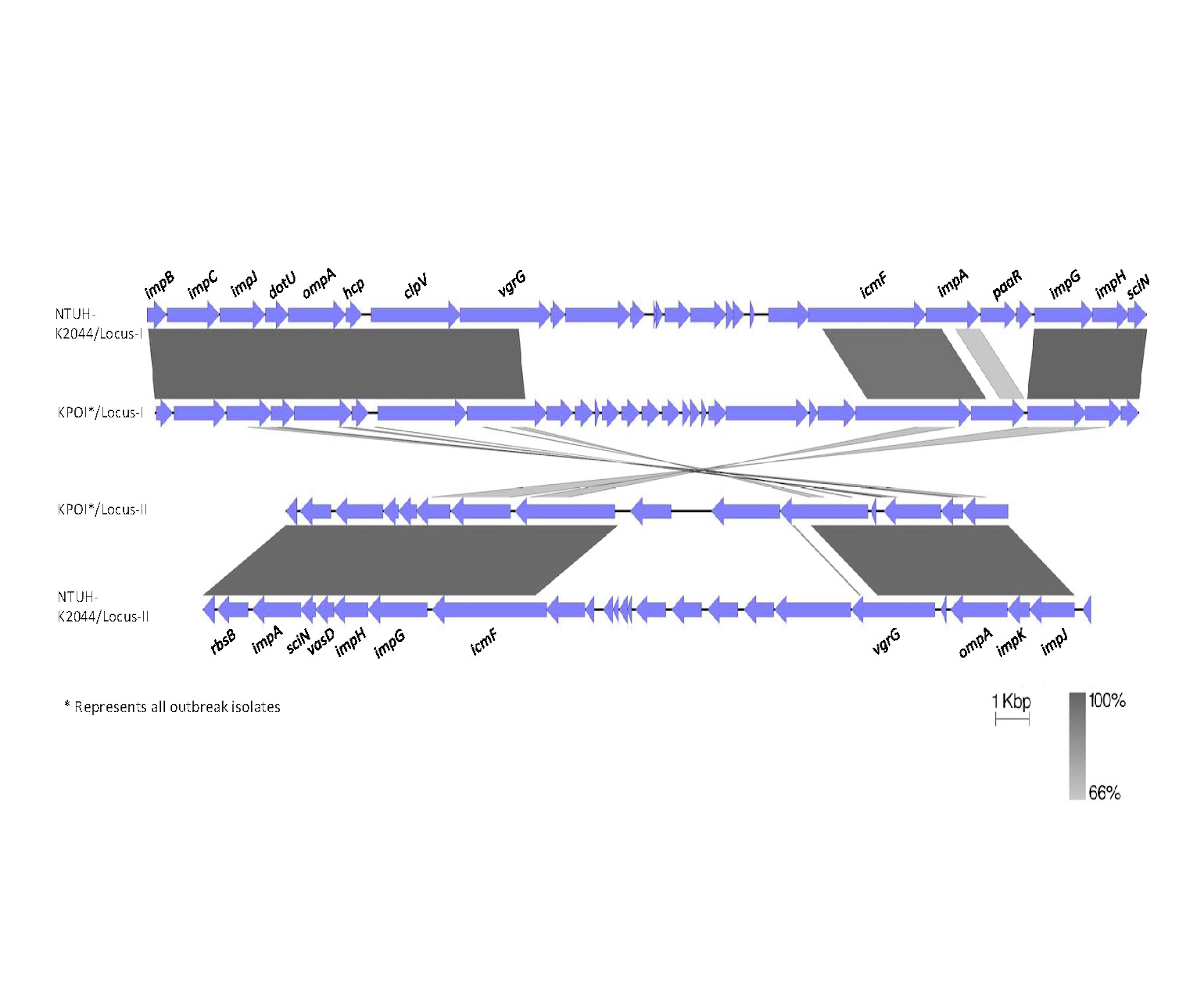

Supplement: Figure S2 — Comparison of the T6SS loci identified in the outbreak clone and NTUH-K2044. The gradients (dark to pale) of the alignment region represent the percentage of sequence identity between samples defined by BLASTn. The clpV gene of NTUH-K2044-Locus II and KPOI-Locus II is not shown in the figure since it is present on a different locus. [file Image2.JPEG]

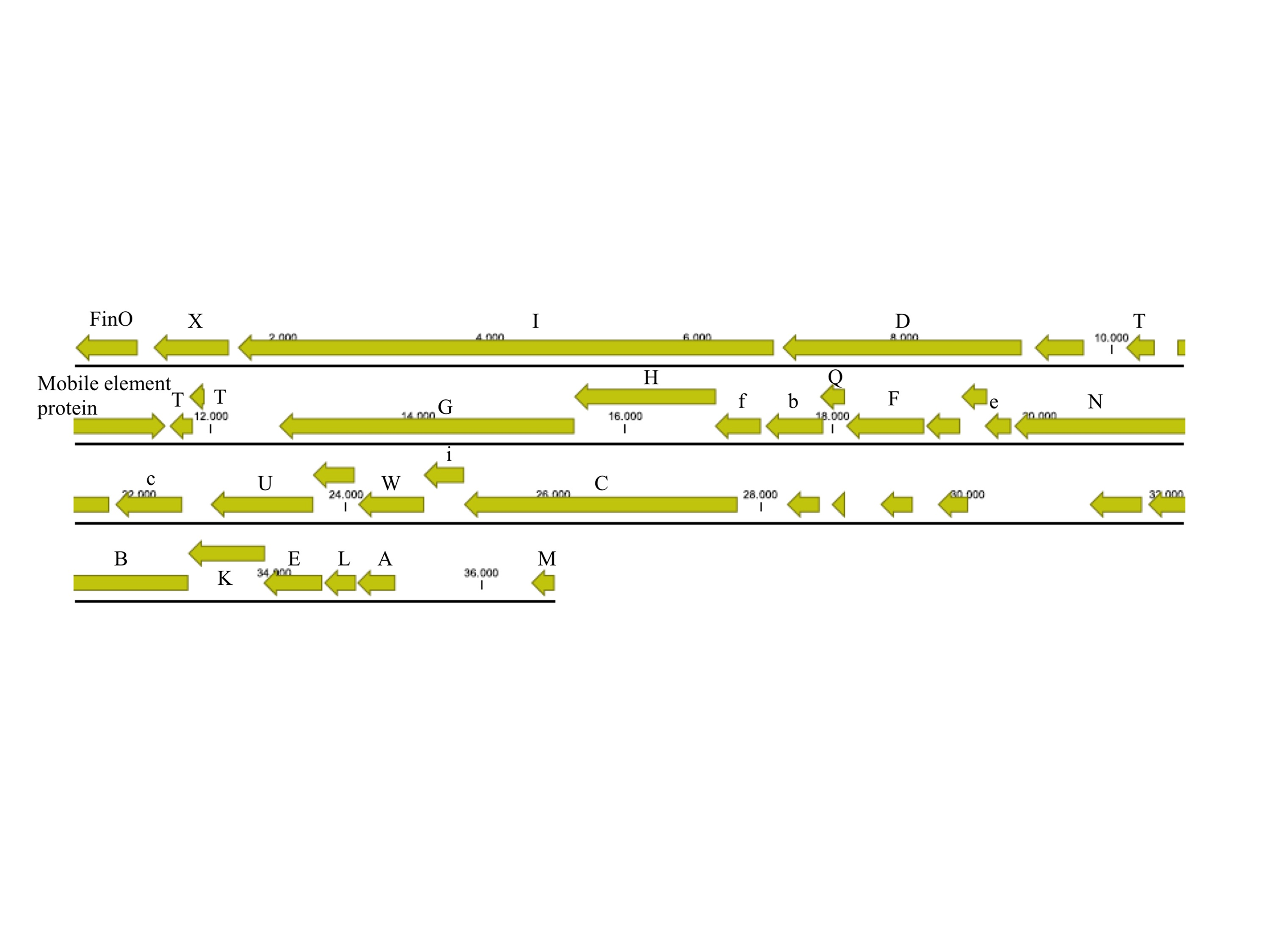

Supplement: Figure S3 — The tra region of the T6SS loci identified in the outbreak clone. The upper cases represent Tra proteins (e.g., A, TraA; B, TraB), and the lower cases represent Trb proteins (e.g., c, TrbC; f, TrbF). [file Image3.TIF]
